# The Selectivity and Specificity of Autophagy in *Drosophila*

**DOI:** 10.3390/cells1030248

**Published:** 2012-06-29

**Authors:** Ioannis P. Nezis

**Affiliations:** 1 Centre for Cancer Biomedicine, Faculty of Medicine, University of Oslo, Montebello, N-0310 Oslo, Norway; Email: ioannis.nezis@rr-research.no; Tel.: +47-2278-1921; Fax: +47-2278-1845; 2 Department of Biochemistry, Institute for Cancer Research, Oslo University Hospital, Montebello, N-0310 Oslo, Norway

**Keywords:** autophagy, development, *Drosophila*, p62, Ref(2)P, programmed cell death, selective autophagy receptors

## Abstract

Autophagy is a process of cellular self-degradation and is a major pathway for elimination of cytoplasmic material by the lysosomes. Autophagy is responsible for the degradation of damaged organelles and protein aggregates and therefore plays a significant role in cellular homeostasis. Despite the initial belief that autophagy is a nonselective bulk process, there is growing evidence during the last years that sequestration and degradation of cellular material by autophagy can be accomplished in a selective and specific manner. Given the role of autophagy and selective autophagy in several disease related processes such as tumorigenesis, neurodegeneration and infections, it is very important to dissect the molecular mechanisms of selective autophagy, in the context of the system and the organism. An excellent genetically tractable model organism to study autophagy is *Drosophila*, which appears to have a highly conserved autophagic machinery compared with mammals. However, the mechanisms of selective autophagy in *Drosophila* have been largely unexplored. The aim of this review is to summarize recent discoveries about the selectivity of autophagy in *Drosophila*.

## 1. Introduction

Autophagy [derived from the Greek words *auto-* (self) and *phagy-* (eating)] is an evolutionarily conserved process where the cells degrade their own cellular material. There are various types of autophagy such as macroautophagy, microautophagy and chaperone-mediated autophagy (for a comprehensive review see [1]). In this review, I will focus on macroautophagy, which from hereafter is referred to as autophagy. During autophagy, there is sequestration of cellular material into double‑membrane vesicles called autophagosomes. The autophagosomes fuse with endocytic vesicles to form the amphisomes, which contain both endocytic and autophagic cargo. The autophagosomes and/or amphisomes are subsequently fused with the lysosomes where the sequestered cargoes are degraded by lysosomal hydrolases. The products of degradation are transported back into the cytoplasm through lysosomal membrane permeases and can be reused by the cell [2].

It was initially considered that sequestration of cytoplasmic cargo is nonspecific. Nevertheless, recent evidence suggests that sequestration of proteins, protein aggregates, organelles and bacteria can be selective through adaptor proteins [3]. Autophagy’s obvious function is the cellular response in nutrient starvation but it is also involved in the removal of damaged organelles, aggregated proteins, and developmental remodeling and therefore plays a major role in cellular homeostasis and development [4,5]. Autophagy is implicated in tumor suppression and progression, neurodegeneration, myopathies, lung and heart disease, diabetes, infections, and obesity [5]. It is therefore very important to elucidate the mechanisms of selective autophagy in normal and pathological conditions. In order to achieve this, it is critical to study the molecular and cellular pathways of selective autophagy in the context of the cell and the system by using living model organisms. The fruit fly *Drosophila melanogaster* is an excellent genetically tractable model organism for investigating selective autophagy. However, the mechanisms of selective autophagy in *Drosophila* have been largely unexplored. This review will summarize the current knowledge about selective autophagy in *Drosophila*.

## 2. Selective Autophagy in *Drosophila*

The various components of the autophagic machinery are very well conserved in *Drosophila* compared with yeast and mammals [6]. However, selective types of autophagy like mitophagy (selective autophagy of mitochondria), xenophagy (selective autophagy of bacteria and viruses), nucleophagy (selective autophagy of nucleus), pexophagy (selective autophagy of peroxisomes), aggrephagy (selective autophagy of protein aggregates), lipophagy (selective autophagy of lipid droplets), ribophagy (selective autophagy of ribosomes) and reticulophagy (selective autophagy of endoplasmic reticulum) are largely unexplored in *Drosophila.* Additionally, the molecular mechanisms of selective sequestration of cargo material and the presence of selective autophagy receptors are poorly described. In the following text I will describe what is known so far about selective autophagy in *Drosophila.*

### 2.1. Selective Degradation of Proteins in Drosophila

In yeast and mammals, autophagy has been shown to preferentially degrade specific proteins like acetaldehyde dehydrogenase [7], catalase [8] and alpha-synuclein [9]. Recently there is evidence that this is also the case for *Drosophila* where there is a growing number of proteins that are specifically degraded by autophagy during various developmental processes.

#### 2.1.1. Degradation of Survival Factors by Autophagy: Shaping Development by Inducing Cell Death in a Restrictive Spatiotemporal Manner

Death of various types of cells is required for normal development [6]. Spatiotemporal regulation of the cell death machinery is very important for this. Recent reports in *Drosophila* shed light on the role of autophagy in the degradation of survival factors which is a delicate way for a cell to die [10].

In the first report we have studied the role of autophagy during oogenesis in *Drosophila melanogaster* [11]. During late oogenesis in the *Drosophila* egg chamber, the nurse cells die after fulfilling their nutritive role to the developing oocyte. Nurse cells exhibit several markers of apoptosis such as caspase activation, chromatin condensation and DNA fragmentation [12]. Using transgenic flies expressing a tandem GFP-mCherry-DrAtg8a transgene and ultrastructural analysis, we have found that autophagy occurs during nurse cell death during the developmental stages 12 and 13 of egg chamber development [11]. To examine the role of autophagy in nurse cell death during late oogenesis, we generated germline mutant cells for the core *Drosophila* autophagy genes *atg1*, *atg13* and *vps34* and scored for cell death using the TUNEL assay in order to detect fragmented DNA. It is worth mentioning that germline clones were generated using the FLP/FRT/ovoD system; the only widely used genetically controlled system to generate germline clones in *Drosophila* [13]. Furthermore, the FLP/FRT/ovoD system was used under conditions where less than 20% of the egg chambers have large follicle cell clones [14]. We found that in autophagy germline mutants, there was a significantly increase in the number of stage 14 egg chambers that had persisting TUNEL-negative nurse cell nuclei. This is also observed in *atg8a* full mutant egg chambers [15]. We also found that cleaved caspase-3 levels were significantly attenuated in autophagy germline mutants compared to wild type. These data show that autophagy is required for nurse cell death and degradation during late oogenesis in *Drosophila melanogaster* and that autophagy functions upstream of caspase processing and DNA fragmentation. Importantly, we showed that the *Drosophila* inhibitor of apoptosis protein, dBruce, colocalized with the autophagosomal marker Atg8a-GFP during late oogenesis and accumulated into large aggregates in *atg1*, *atg13* and *vps34* germline mutants. These observations showed that dBruce is degraded by autophagy in the nurse cells during late oogenesis. Additionally, *atg1* and *dBruce^E81^* or *vps34* and *dBruce^E81^* double mutant egg chambers contained persistent nurse cell nuclei that were TUNEL-positive. Taken together, these data indicate that dBruce is required for nurse cells survival during oogenesis in *Drosophila* by controlling DNA fragmentation [11]. Autophagic degradation of dBruce is not dependent on the autophagy receptor Ref(2)P [11,16] (see also below), even though proteolytic fragments of dBruce (~140 and ~80 kD) are ubiquitinated [17]. These fragments could be targeted to the autophagic machinery via other yet unknown autophagy receptors. An alternative scenario could be that dBruce may bind directly to autophagosomal membranes via a LIR (LC3 interacting region) motif as found for other selective autophagy receptors (see below). As such, dBruce would be a selective autophagy substrate and ubiquitination would not be needed for its degradation. A bioinformatics search of the dBruce amino acid sequence revealed the presence of 15 putative LIR motifs and this is an issue that needs further investigation.

McCall and colleagues showed that dICAD and DNaseII participate in nurse cells death in late *Drosophila* oogenesis and that there was a significant decrease in TUNEL positive staining in dICAD mutants. They also showed that dying nurse cells exhibit signs of programmed necrosis and that genes encoding proteins involved in lysosome biogenesis or function, like *dor*, *spinster* and *cathepsin D* are required for this process [18]. Additionally *atg8a* was found to act as an enhancer of *spinster*, further supporting the requirement of autophagy genes for cell death [19]. dBruce was shown to have an important role during sperm individualization where caspase activity must be tightly regulated and restricted to a particular region of the cell [20]. *Drosophila* oogenesis is a defined developmental process where the nurse cells are sacrificed for their sister cell, the oocyte, after they have provided the necessary nutrients for future embryonic development [21]. Nurse cell death must be very carefully controlled in order to prevent oocyte destruction from lethal caspase activity. We propose that dBruce is a critical regulator of cell death during *Drosophila* oogenesis. We suggest that autophagic degradation of dBruce promotes limited activation of drICE that can lead to dCAD-mediated TUNEL‑detectable DNA fragmentation and subsequently DNaseII can function to degrade nurse cell DNA further in autolysosomes. In conclusion, our findings indicate that autophagy plays an important role in nurse cell death during late oogenesis in *Drosophila*, first by acting upstream of DNA fragmentation thereby causing cell death, and then by removing nurse cell remnants [11].

In the second report, Jan and colleagues showed that inhibitor of apoptosis protein DIAP1 is degraded during dendrite pruning of *Drosophila* class IV dendritic arborization (da) neurons. This is depended on Valosin-containing protein (VCP), a ubiquitin-selective AAA chaperone involved in endoplasmic reticulum-associated degradation and the maturation of autophagosomes [22,23]. The authors demonstrated that *Drosophila* VCP is required for proper dendrite morphology and viability of *Drosophila* larval da neurons and that VCP is required for apoptosis and neuronal remodeling. Interestingly, inhibition of VCP causes defects in caspase activation and degradation of the caspase inhibitor DIAP1, whereas VCP was shown to be required for DIAP1 degradation in *Drosophila* S2 cells [22]. DIAP1 contains three major structural motifs: two baculovirus inhibitor of apoptosis repeat (BIR) domains which are required for interactions with caspases and the RHG proteins Reaper, Hid and Grim; and a C-terminal RING finger domain that is required for the ubiquitylation of bound caspases and for the auto-ubiquitylation of DIAP1 under apoptosis-inducing conditions. Biochemical analysis showed that an intact BIR1 domain is a major determinant for VCP binding [22]. Taken together, the above results showed that degradation of DIAP1 during the fine developmental process of dendrite pruning is controlled by the autophagy regulator VCP. However, the molecular details of selective sequestration of DIAP1 need to be further clarified. These results suggest that autophagic degradation of survival factors can cause cell death during development in *Drosophila*. 

It is worth mentioning that autophagy has also a pro-survival function in differentiated cells like neurons. In *Drosophila* photoreceptors the inhibition of autophagy leads to cell death [24]. Edgar and colleagues showed that hyperactivation of TOR (target of rapamycin) leads to photoreceptor cell death in an age- and light-dependent manner and that this is because of TOR’s ability to suppress autophagy. They also showed that genetic inhibition of TOR or inducing autophagy suppresses cell death in *Drosophila* models of Huntington’s disease [24]. Autophagy observed in Atrophin-1 mutants also leads to cell death [25]. Furthermore, in a very interesting study, Molllereau and collegues showed that mild ER stress is neuroprotective in *Drosophila* models of Parkinson disease. They found that the combination of mild ER stress and apoptotic signals triggers an autophagic response both *in vivo* and *in vitro* and provide evidence that autophagy inhibits caspase activation and apoptosis [26]. Together these data indicate that autophagy alleviates cell death in several common types of neurodegenerative diseases and that its role in cell death is context dependent. It appears that developing tissues are more prone to induce autophagic cell death than differentiated cells in which autophagy has a pro-survival function.

#### 2.1.2. Retinal Degeneration and Degradation of Rhodopsin

Autophagic degradation of proteins was also shown to be important in another context of development; this is the case of retinal degeneration. It is known in *Drosophila* that activated rhodopsin is degraded in endosomal pathways in normal photoreceptor cells and that accumulation of activated rhodopsin in some mutants leads to retinal degeneration [27]. In a recent study it was shown that autophagy is responsible for the degradation of activated rhodopsin in order to prevent retinal degeneration [28]. Knock-down or mutation of autophagy genes, such as autophagy-related protein 7 and 8, or genes essential for PE (phosphatidylethanolamine) biogenesis and autophagosome formation, including phosphatidylserine decarboxylase (Psd) and CDP-ethanolamine:diacylglycerol ethanolaminephosphotransferase (Ept), caused light-dependent retinal degeneration in the *Drosophila* eye. Silencing of atg-7/8 or Psd/Ept resulted in an increase in the amount of rhodopsin localized to Rab7-positive late endosomes [28]. These results suggest that autophagic and endosomal/lysosomal pathways suppress light-dependent retinal degeneration and that rhodopsin is a substrate for autophagic degradation in this context. However, it is not clear which molecular mechanisms are required for the degradation of rhodopsin and how this is regulated *versus* bulk degradation.

#### 2.1.3. Degradation of Highwire

In addition to its roles in cellular homeostasis, autophagy is vital in a range of physiological contexts during developmental growth and remodeling of various tissues during development [4,5]. One such example in *Drosophila* is the development of synapses in the larval neuromuscular junction. It was shown that autophagy promotes the development of synapses in the larval neuromuscular junction, by downregulating Highwire, an E3 ubiquitin ligase, which limits neuromuscular junction growth [29,30]. Atg1 overexpression resulted in neuromuscular junction overgrowth and this was suppressed by mutations in atg18, suggesting that this overgrowth is due to elevated levels of autophagy [29,30]. Moreover, mutations in autophagy genes caused neuromuscular junction undergrowth. In a recent paper, Wu and colleagues, using tandem affinity purification and liquid chromatography-mass spectrometry, identified *Drosophila* Rae1 as a component of the Highwire complex. Rae1 mutant flies exhibited morphological defects at the neuromuscular junction, a phenotype that mimics that observed in Highwire mutants [31]. Rae1 was shown to genetically and physically interact with Highwire and to restrain synaptic growth by regulating the MAP kinase kinase kinase Wallenda. Moreover, it was shown that Rae1 binds to Highwire and protects it from autophagic degradation [31]. Together, these findings demonstrate that Rae1 prevents degradation of Highwire by autophagy and that selectively controls Highwire protein abundance during synaptic development.

### 2.2. Mitophagy, Xenophagy and Nucleophagy in Drosophila

Selective autophagy has an important role in the quality control of organelles and intracellular pathogens [32,33]. However, mitophagy (selective autophagy of mitochondria), xenophagy (selective autophagy of bacteria and viruses) and nucleophagy (selective autophagy of nuclear fragments) are largely unexplored in *Drosophila*. Moreover, pexophagy (selective autophagy of peroxisomes), lipophagy (selective autophagy of lipid droplets), ribophagy (selective autophagy of ribosomes) and reticulophagy (selective autophagy of endoplasmic reticulum) are not described yet in *Drosophila*. In the following text I will summarize what is reported so far in the literature about the processes above in *Drosophila*.

#### 2.2.1. Mitophagy

Selective autophagic degradation of mitochondria has been recently described in yeast and mammals and attracted great attention because of its association in various human diseases like neurodegeneration and myopathies [32]. Studies in yeast showed that the outer mitochondrial membrane protein Atg32 binds to the autophagosomal membrane protein Atg8 through its LIR motif and mediates mitophagy [34]. In mammalian cells, the kinase PTEN-induced putative kinase protein 1 (PINK1) accumulates in damaged miitochondria and was shown to recruit the E3 ubiquitin ligase Parkin from the cytoplasm specifically to the damaged mitochondria. Parkin ubiquitylates mitochondrial proteins and promotes mitochondrial degradation by autophagy [32]. Additionally, in mammals mitophagy has an important role during the physiological process of red blood cell differentiation and it requires the outer mitochondrial membrane protein NIP3-like protein NIX, which also binds to LC3 through its LIR motif [35,36]. 

The PINK1-Parkin pathway in *Drosophila* promotes mitochondrial fission [37,38]. It was reported that in S2 *Drosophila* cells, PINK1 localizes to depolarized mitochondria and recruits Parkin. This in turns promotes mitochondria degradation by autophagy [39]. Interestingly, the profusion factor mitofusin (Mfn; also known as marf in Drosophila) was shown to be a novel substrate of Parkin [39]. It was also shown that activation of autophagy through Atg1 over expression rescues PINK1 mutant phenotypes in *Drosophila* [40]. Mitochondrial dysfunction caused by protein conformational stress was recently shown to induce mitophagy, which is Parkin-dependent [41]. Importantly, the *Drosophila* homologue of the mammalian selective autophagy receptor p62, Ref(2)P (see also below), acts genetically downstream of Parkin to promote the clearance of dysfunctional mitochondria in this context [41]. These studies suggest that the mechanisms of mitophagy in *Drosophila* are well conserved compared with mammals, however the molecular details have to be further clarified.

#### 2.2.2. Xenophagy

Autophagy is an important weapon of cellular defense against intracellular pathogens. Xenophagy, the selective autophagic degradation of bacteria and viruses, is well documented in mammals [33]. However, in *Drosophila* xenophagy is largely unexplored. There are a few studies that provide evidence that the mechanisms of xenophagy are conserved in *Drosophila*. Kurata and colleagues showed that in S2 cells and primary *Drosophila* hemocytes, *Listeria monocytogenes* intracellular growth was prevented by autophagy and this was crucial for host cell survival [42]. Recognition of diaminopimelic acid-type peptidoglycan by the pattern-recognition receptor PGRP-LE was important for the induction of autophagy. Importantly, the IMD innate signaling pathway was not required for autophagy induction [42].

It was also found by Cherry and colleagues that autophagy has a protective role against the mammalian viral pathogen Vesicular Stomatitis Virus (VSV) in D*rosophila* S2 cells as well as in adult flies [43]. The pathogen-associated molecular pattern that initiates autophagy was shown to be the surface glycoprotein of VSV, VSVG. VSV-induced autophagy was found to be dependent on Toll-7 in S2 cells as well as in adult flies [43,44]. Autophagy restrains viral replication, and inhibition of autophagy resulted to increased viral replication and pathogenesis. Importantly, it was found that this response was controlled by the phosphatidylinositol 3-kinase (PI3K)/Akt signaling pathway which controls autophagy in response to nutrient availability [43]. These data suggest that xenophagy occurs in *Drosophila* and the molecular mechanisms are well conserved compared with mammals.

#### 2.2.3. Nucleophagy

Nucleophagy is the selective autophagic degradation of the nucleus or parts of the nucleus [45]. It has been described in filamentous fungi [46], in *Tetrahymena thermophila* [47] and in yeast [48]. This process is well described in yeast *Saccharomyces cerevisiae*, and is called piecemeal microautophagy [48]. During piecemeal microautophagy parts of the nucleus are sequestered into invaginations of the vacuolar membrane, followed by fission of nuclear fragments and its release into the vacuolar lumen, where they are degraded [45,48]. Nucleophagy in mammals was described in nuclear envelopathies caused by mutations in the genes encoding A-type lamins (LMNA) and emerin (EMD) [49]. Nucleophagy was also observed rarely in wild-type cells [49]. Additionally, it has been recently reported that micronuclei in mammalian cells in culture can be removed by autophagy [50].

Nuclear autophagy has been recently described in *Drosophila* egg chamber during the cell death of nurse cells in late oogenesis [11]. Specific expression of the autophagic marker mCherry-DrAtg8a in the nurse cells during the late stages of oogenesis, showed the presence of large autolysosomes attached to or adjacent to the condensed and fragmented nurse cell nuclei. Ultrastructural analysis revealed the presence of large autolysosomes which contained condensed material resembling the material of the fragmented nurse cell nucleus, suggesting that the nurse cell nucleus is removed by autophagy [11].

### 2.3. Selective Autophagy Receptors in Drosophila

#### 2.3.1. Refractory to Sigma P (Ref(2)P), the Drosophila Orthologue of Mammalian Selective Autophagy Receptor p62/SQSTM1

Selective autophagy receptors are under intensive examination in mammals, and six proteins have been identified so far: p62/SQSTM1, NBR1, NDP52, Nix, optineurin and Stbd1 [3,51,52]. All these proteins contain a LIR (LC3 -interacting region) motif and have been shown to physically interact with the autophagosomal membrane protein LC3 (microtubule-associated protein 1 light chain 3) [3].

Landmark studies from Johansen’s group showed that mammalian p62/SQSTM1 is a selective substrate for autophagic degradation [53,54]. p62/SQSTM1 is the first identified and most studied selective autophagy receptor. It is a multifunctional scaffold protein that serves a large variety of cellular functions [3,55,56]. The human p62 protein contains several structural and functional motifs [3], including a Phox and Bem1p domain (PB1 domain) located at the N-terminus, a zinc finger-type (ZZ-type) domain, a TRAF6 binding (TB), nuclear localization signals (NLSs) and nuclear export signal (NES), a LC3-interacting region (LIR motif) and a kelch-like ECH-associated protein 1 (KEAP1) interacting region (KIR) motif responsible for the interaction with LC3 and KEAP1, respectively [3]. The C-terminus of p62 harbors a ubiquitin-associated (UBA) domain required for its binding to mono- and poly-ubiquitin [3].

The *Drosophila* single p62 homologue, Ref(2)P, (*refractory to Sigma Pref(2)P/CG10360*), has an N-terminal PB1 domain followed by a ZZ-type zinc finger domain and a C-terminal UBA domain [16,56]. There are several lines of evidence that support the hypothesis that Ref(2)P is a selective autophagy receptor. It has been shown that Ref(2)P is a major component of protein aggregates formed during normal aging in *Drosophila* adult brain, of protein aggregates in flies that are defective in autophagy, in flies that have impaired proteasomal function and in *Drosophila* models of human neurodegenerative diseases [16,57]. The abilities of Ref(2)P to bind ubiquitinated proteins (through its UBA domain) and to multimerize (through its PB1 domain) are required during the *in vivo* formation of protein aggregates in the adult brain of *Drosophila* [16]. Additionally, it was recently shown that Ref(2)P has a role in selective autophagic degradation of ubiquitinated proteins which are accumulated following the deubiquitinating enzyme dUsp36 inactivation [58].

Importantly, bioinformatics analysis of the sequence of Ref(2)P revealed the presence of a putative LIR motif. The human p62 LIR motif is a 22 amino acid-long sequence which contains an evolutionarily conserved motif of three acidic residues followed by a tryptophan (DDDW in p62) [3]. Sequence logo from 25 different LIR motifs from 21 different proteins that all have been tested for binding to ATG8 family proteins revealed that the LIR motif is 7–9 amino acids long and has the following consensus motif: D/E-D/E-D/E-W/F/Y-X-X-L/I/V. There is a requirement for aromatic residues in the W-site (W/F/Y) and also a requirement for large, hydrophobic residues in the L-site (L/I/V) [3,59]. Bioinformatics analysis of Ref(2)P sequence revealed the presence of a putative LIR between amino acids 451-458 with a sequence DPEWQLID, which fits very well with the criteria for aromatic residues at W site (W) and hydrophobic residues at L site (I). The functional roles of putative LIR motif of Ref(2)P have to be tested experimentally *in vitro* and in *vivo*. Taken together the above information suggests that Ref(2)P is a selective autophagy cargo receptor in *Drosophila melanogaster*.

Ref(2)P was initially characterized in a screen for modifiers of sigma virus multiplication [60,61,62]. There are restrictive alleles of ref(2)P which reduce the replication of sigma virus and permissive alleles which allow efficient multiplication of the virus [60,63,64]. In flies having the permissive alleles the probability of infection may reach 100%, whereas in flies with restrictive alleles the infection rate drops to 0.01%, at least for some viral strains [63,64]. Homozygous Ref(2)P null flies are fully viable but the males are sterile. The molecular and cellular mechanisms of male sterility are not clear [60,61]. The testes of ref(2)P^od1^and ref(2)P^od3^ loss-of-function mutants (where Ref(2)P protein lacks the UBA domain) and ref(2)P^od2^ loss-of-function mutant (where Ref(2)P protein lacks the PB1 domain), exhibit morphological characteristics of degeneration, such as the appearance of large myelin figures around the spermatids [61]. Additionally, the mitochondria appeared degenerated [61]. 

One intriguing question is how Ref(2)P controls sigma virus multiplication at the molecular and cellular level. It was previously shown that there is a direct interaction between Ref(2)P and the sigma virus capsid P protein [65]. Ref(2)P also shares conformation dependent epitopes with the capsid N protein [65]. Additionally, Ref(2)P has been shown to interact genetically with DaPKC and the *Drosophila* homologue of TRAF6, dTRAF2, to participate in the Toll-signaling pathway, and to regulate the NF-kB proteins Dorsal and DIF [66,67]. Interestingly, mammalian p62 was shown to interact with sindbis virus capsid protein, and knockdown of p62 blocked the sequestration of sindbis capsid to autophagosomes [68]. Taken together these results suggest that Ref(2)P may target sigma virus capsid for autophagosomal degradation and also may function as a scaffolding protein during assembly of viral protein complexes. 

Another aspect of Ref(2)P function was recently reported in *Drosophila* hemocytes. Ref(2)P was shown to control hemocyte spreading and protrusion formation [69]. This observation suggests that selective autophagy of an ubiquitinated substrate may function in an autophagy-dependent mechanism for cortical remodeling of hemocytes. Taken together all the above information demonstrates that Ref(2)P, like its mammalian homologue p62, has diverse cellular functions whose molecular mechanisms have to be further examined .

#### 2.3.2. Bluecheese, the *Drosophila* Homologue of the Mammalian Selective Aggregate Clearance Mediator Alfy

The mammalian phosphatidylinositol-3-phosphate (PI3P)-binding protein Alfy is a selective aggregate clearance regulator [70,71,72]. It contains several functional motifs including a BEACH domain followed by a series of WD40 repeats and a PI(3)P-binding FYVE domain [70]. Alfy is not found on endosomes but instead localizes mainly to the nuclear envelope. Under conditions of starvation Alfy relocalizes to cytoplasmic structures located close to autophagic membranes and ubiquitin-containing protein aggregates. Alfy is required for selective degradation of aggregated proteins such as polyQ-containing mutant huntingtin [71]. This function was proposed to be mediated by Alfy’s physical interaction with Atg5, PI(3)P and p62 [71,72]. Therefore Alfy functions as a scaffold protein for recruitment of misfolded, ubiquitinated proteins to the autophagosomal membrane that become degraded by autophagy.

Bluecheese is the *Drosophila* homologue of Alfy and is highly conserved to its human homologue (~50% identity between fly and human homologs) [70,73] and it contains similar functional domains at its C-terminal. *blue cheese* mutant flies exhibit a reduced adult life span and age-related neurodegeneration associated with accumulation of ubiquitin-conjugated protein aggregates throughout the adult central neurous system and cell death [73]. Ref(2)P accumulates in ubiquitinated inclusions in the brain of *blue cheese* mutant flies, suggesting that Bluecheese is required for autophagic degradation of p62-associated ubiquitinated proteins *in vivo* [72].

It was recently shown by Simonsen group that overexpression of the C-terminal region of Bluecheese ameliorates neurodegeneration related phenotypes *in vivo* [71]. Enhanced expression of full‑length Bluecheese (UAS-FL-Bchs) or C-terminal Bluecheese (UAS-bchs-C1000) with UAS‑polyQ127 in the eye resulted in a reduced number of necrotic areas and an overall improvement in eye size, morphology, and pigmentation. Taken together, these results suggest that the Alfy/Bchs proteins have a role in macroautophagic clearance of aggregation-prone proteins.

## 3. Conclusions

The molecular and cellular mechanisms of selective autophagy in *Drosophila* remain largely unexplored. The detailed mechanisms of selective autophagy of organelles and proteins has not been directly shown in *Drosophila*, and the molecular details of the interaction of selective autophagy receptors Ref(2)P and Bluecheese with the autophagic machinery have to been shown experimentally. The presence of a putative LIR motif in Ref(2)P offers a fertile ground for further functional analysis *in vivo*. p62 and Ref(2)P have been proposed to collect ubiquitinated proteins and target them for degradation [3]. It would therefore be interesting to test whether induced expression of Ref(2)P ameliorates phenotypes related to neurodegeneration *in vivo*. Elucidation of the mechanisms of selective autophagy may have applications in fighting aggregation-related diseases, such as neurodegenerative diseases as well as cancer. 

In conclusion, *Drosophila* offers a fertile ground for studying the molecular and cellular mechanisms of selective autophagy. Future studies will hopefully uncover the molecular details of this fascinating process.
